# Optimizing growth efficiency and energy economics in vertical farming through dynamic reduction of blue light in lettuce baby leaf (*Lactuca sativa L*.)

**DOI:** 10.3389/fpls.2026.1827422

**Published:** 2026-04-22

**Authors:** Vivek Jadhav, Vito Aurelio Cerasola, Karin Nilsson, Marco Ghio, Michael Martin, Giuseppina Pennisi, Giorgio Gianquinto, Francesco Orsini

**Affiliations:** 1 Department of Agricultural and Food Science (DISTAL),Alma Mater Studiorum – University of Bologna, Bologna, Italy; 2 Nära Sverige AB, Stockholm, Sweden; 3 Germina S.r.l., Genova, Italy; 4 Division of Life Cycle Management, Department of Sustainable Society, IVL Swedish Environmental Research Institute, Stockholm, Sweden; 5 Department of Sustainable Development, Environmental Science and Engineering, KTH Royal Institute of Technology, Stockholm, Sweden

**Keywords:** artificial lighting, dynamic lighting, economic efficiency, energy optimization, growth patterns, indoor cultivation

## Abstract

**Introduction:**

Vertical farming offers a sustainable solution for urban food production, but energy optimization remains a critical challenge, with nearly half of the electricity requirements dedicated to artificial lighting. Dynamic adjustment of blue and red light can reduce energy costs, as blue light is more energy-intensive, thereby lowering operating expenses and increasing profitability.

**Methods:**

This research investigates the effects of dynamic adjustment of blue and red light on lettuce (*Lactuca sativa*, cv. Danstar) plants. Four light treatments were tested, each maintaining a total photosynthetic photon flux density (PPFD) of 200 μmol m^-2^ s^-1^ under a 16-hour photoperiod: (1) RB3 (control, 150 μmol m^-2^ s^-1^ red and 50 μmol m^-2^ s^-1^ blue); (2) 25% blue (B) reduction with hourly alternation between control and 175 μmol m^-2^ s^-1^ red/25 μmol m^-2^ s^-1^ blue; (3) 38% B reduction with hourly cycling through RB3, 162/38, 175/25, and 188/12 μmol m^-2^ s^-1^ of red/blue light; and (4) 50% B reduction with hourly alternation between control and 200 μmol m^-2^ s^-1^ monochromatic red. Agronomical, physiological, and morphological data were collected weekly from 7, 14, and 21 days after transplanting.

**Results:**

While the 50% B dynamic treatment did not enhance overall crop performance compared to the RB3 control, 25% B and 38% B increased lettuce fresh yield by 50-60%, with dry weight remaining stable.

**Discussion:**

These responses indicate improved leaf hydration (reduced dry matter content) resulting in increased fresh marketable yield, improved light-energy use efficiency by up to 63% and reduced lighting costs by 40%, demonstrating that constant blue light at a fixed PPFD is not required for optimal growth. This approach may offer a viable strategy to reduce production costs and enhance sustainability in controlled environment agriculture.

## 1 Introduction

Global food security faces escalating challenges driven by limited arable land, resource depletion, water scarcity, and increasing climatic variability (Malhi et al., 2021; Carotti et al., 2023). Concurrently, the continuous trend of urbanization and rapid population growth poses a significant challenge, with an expected 68% of the global population living in urban areas by 2050, when the world population is projected to reach 9.5 billion, placing massive pressure on the food supply chain (United Nations Department of Economic and Social Affairs (UN DESA), 2018). Together, these challenges are driving the development of innovative and sustainable agricultural systems that bring food production closer to consumers, fostering robust, adaptive, and resource-efficient farming approaches that ensure stable yields while minimizing environmental impact (Martin et al., 2023).

Vertical farming systems (VFs) are indoor agricultural facilities where crops are grown in multiple stacked layers within controlled environments. These systems allow for year-round production, especially in urban areas where space and resources availability limit conventional farming (Ji et al., 2023). By regulating environmental factors such as light, temperature, CO_2_, and nutrients, VFs optimize plant growth and resource use efficiency (primarily water, land, and fertilizers), reducing food miles and pesticide use (Pennisi et al., 2025). Despite these benefits, high energy and operational costs remain significant challenges in VFs, highlighting the need for improved environmental control strategies to maximize yield and quality with minimal energy input.

Light is a key environmental factor driving plant growth and development, providing the energy source for photosynthesis and regulating several physiological processes through photoreceptors such as phytochromes, cryptochromes, and phototropins (Vaštakaitė-Kairienė et al., 2022; Chen et al., 2021; Trivellini et al., 2023). Recent advances in LED technology have enabled precise control of light quality, intensity, and duration, enhanced crop productivity and enabling detailed studies of plant responses to specific spectra (Kusuma et al., 2020; Son and Oh, 2015). Lighting efficacy, measured as photosynthetic photon flux per unit of electrical power, is strongly influenced by spectral quality (Kusuma et al., 2020). Blue (450 nm) and red (660 nm) photons differ in both energy consumption and photosynthetic efficiency, with blue photons requiring more energy and being approximately 25% less efficient for photosynthesis than red photons (Mitchell and Sheibani, 2020). Red and blue light are widely recognized for their positive effects on plant growth: while blue light promotes compact plant architecture and enhances photosynthetic pigment synthesis, monochromatic red light stimulates cell elongation (Silva et al., 2024). However, neither monochromatic red nor blue light alone supports optimal plant growth, and a combined red-blue spectrum generally ensures superior growth performance. For instance, monochromatic red light can cause abnormal leaf morphology and reduced photosynthesis (Wang et al., 2016), while blue light is essential for photomorphogenesis, including stomatal regulation and chloroplast development (Wang et al., 2015). The application of blue light also affects nutritional quality by increasing anthocyanin and carotenoid accumulation in lettuce (Paradiso and Proietti, 2022). Studies have shown that even a small proportion of blue light (e.g., 7% of total photosynthetically active radiation) is necessary to maintain efficient photosynthesis and prevent dysfunctions in cucumber (Hogewoning et al., 2010). Contrarily, high levels of blue light can delay the ripening of pepper fruit (Liu et al., 2022) and activate stress and secondary metabolite pathways in *Centella asiatica* (Nawae et al., 2021).

Variations in the proportion of blue light significantly affects plant biomass, morphology, and energy consumption (Chen et al., 2021; Wang et al., 2024a), although the effects of light quality on plant growth and phytochemical composition vary across species and developmental stages, making crop-specific light optimization essential (Yuan et al., 2025). Previous studies on lettuce have demonstrated that a red-to-blue (RB) light ratio of 3 optimizes yield, enhances leaf quality, and improves energy and water use efficiency, emphasizing the critical role of spectral composition in controlled environment cultivation (Pennisi et al., 2019). Also, Zauli et al. (2024) evaluated RB ratios of 3, 5, 7, and 9 in baby-leaf kale (*Brassica oleracea*), identifying the RB ratio of 5 as the most effective spectrum for enhanced production and highlighting that species-specific responses to RB ratios also occur.

While VFs often operate under constant conditions, dynamic lighting strategies that adjust blue light intensity in response to plant developmental stage and environmental factors can improve energy efficiency without compromising growth (Kaiser et al., 2024). In natural environments, plants experience dynamic changes in light intensity and spectral quality, driven by factors such as cloud cover, canopy movement, and shading. Plants have evolved adaptive photo-regulatory responses to these fluctuations (especially to short wavelengths), which influence stomatal behavior, carbon assimilation, and water-use efficiency (Christie, 2007). Building on this natural model, the present study investigates a dynamic lighting strategy that modulates red and blue light intensities hourly to enhance sustainability and resource efficiency in commercial VFs. The aim of this study was to evaluate whether reducing blue light energy input through hourly spectral modulation can maintain or improve plant performance in controlled environments. To do so, lettuce (*Lactuca sativa* cv. Danstar) was chosen because it has long served as a model species in controlled environment and VFs research due to its short growth cycle, compact growth habit, short production cycles (especially when harvested at the baby-leaf stage), and wide use in controlled-environment research (Carotti et al., 2023).

## 2 Materials and method

### 2.1 Plant material and experimental setup

**Figure 1:**
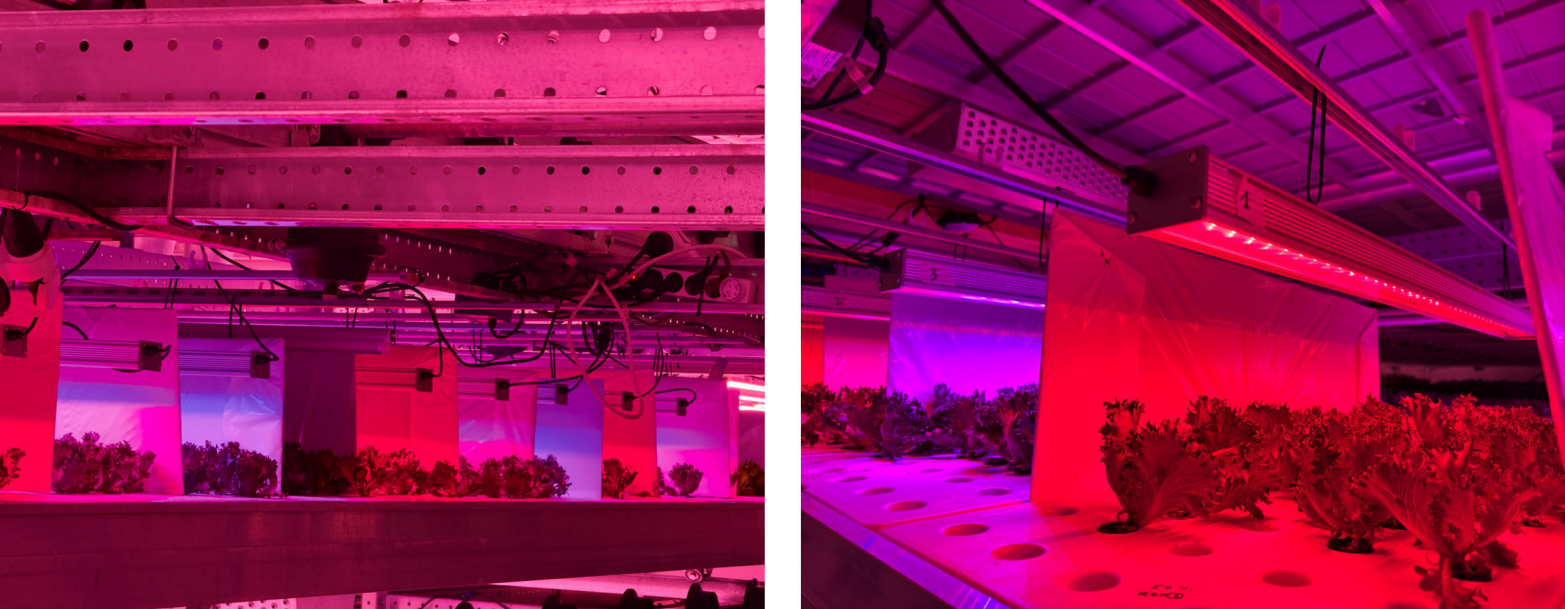
Overview of the experimental setup at Nära Sverige AB, a commercial vertical farm located in Stockholm, Sweden. Twelve light-insulated compartments, each measuring 0.5 m², were constructed for this study.

The experiment was conducted at Nära Sverige AB, a commercial vertical farm in Stockholm, Sweden (**Figure 1**). Lettuce seeds (*Lactuca sativa* cv. Danstar; BASF, Netherlands) were sown in polystyrene trays (60 × 40 × 4 cm; MODIFORM B.V., Netherlands) using Quick Plugs (Quick Plug B.V., Netherlands) and germinated at 98% relative humidity. Seedlings were then transferred to an ebb-and-flow system under full-spectrum LED light (Valoya L28/AP673L) at 100 μmol m^-2^ s^-1^ photosynthetic photon flux density (PPFD) for 12 days. At 14 days after sowing (DAS), seedlings were transplanted into high-density polyethylene (HDPE) hydroponic floating trays (Meteor System, Netherlands) at a density of 140 plants m^-2^ and subjected to four light treatments, as further detailed in Section 2.2 in **Table 1**. A standard Hoagland nutrient solution was prepared with an electrical conductivity (EC) of 2.0 mS cm^-1^ and a pH of 6.0 and circulated for 20 minutes every 2 hours. The nutritional composition was as follow: 14 mM NO_3_^-^-N, 1.0 mM NH_4_^+^-N, 1.0 mM P, 6.0 mM K, 4.0 mM Ca, 2.0 mM Mg, 2.0 mM S, 45 µM Fe, 1.0 µM Cu, 1.0 µM Zn, 45 µM B, 10 µM Mn, 1.0 µM Mo. A closed-loop system with an automated fertigator (Priva NutriJet, Netherlands) adjusted pH and EC twice daily. Environmental conditions were adjusted at 22 °C/19 °C day/night, 60–70% relative humidity, and CO_2_ was enriched at 1200 ppm during the photoperiod (06:00–22:00). For the research, the central portion of an ebb-and-flow cultivation sector was utilized, within which twelve separate compartments (each of 0.5 m^2^ surface, **Figure 1**) were created. Treatments were randomly allocated to compartments, with 3 replicates per compartment, and destructive harvests were conducted at 7, 14, and 21 days after transplanting (DAT) across four lighting treatments to determine plant growth performance.

**Table 1:** Overview of the red and blue light treatments applied in the experiment. 25%B, 38%B, and 50%B indicate the percentage of daily blue light integral reduced as compared to the RB3 control.

Treatment	Red light (µmol m^-2^ s^-1^)	Blue light (µmol m^-2^ s^-1^)	Blue reduction (µmol m^-2^ s^-1^)	Red increase (µmol m^-2^ s^-1^)	Spectral schedule during photoperiod (06:00–22:00)	Total PPFD (µmol m^-2^ s^-1^)	Energy use (kWh m^-2^ d^-1^)
Control (constant)	150	50	–	–	Constant spectrum	200	2.06
25% B (dynamic)	150 175	50 25	- 25	- 25	Hourly alternation between (R/B) 150/50 and 175/25	200	2.03
38% B (dynamic)	150 162 175 188	50 38 25 12	- 12 25 38	- 12 25 38	Stepwise hourly reduction over four periods (R/B): 150/50 → 162/38 → 175/25 → 188/12	200	2.02
50% B (dynamic)	150 200	50 -	- 50	- 50	Hourly alternation between (R/B) 150/50 and 200/0	200	1.98

A graphic representation of the experimental treatments is reported in **Supplementary Figure 1**.

### 2.2 Light treatments

Lighting was provided by an integrated LED panel (Crocus Labs GmbH, Germany), emitting narrow-band red (660 nm) and blue (450 nm) wavelengths. The system enabled precise, independent control of red and blue PPFDs, facilitating dynamic modulation via pre-programmed spectral recipes controlled via customized software. Light treatments were carefully calibrated using a LI-180 spectrometer (LI-COR Biosciences, USA) to maintain total PPFD at 200 ± 5 μmol m^-2^ s^-1^. Four lighting regimes were implemented, as detailed in **Table 1**. Following the lighting protocol developed by Pennisi et al. (2019) for lettuce, RB = 3 (RB3: red 150 μmol m^-2^ s^-1^ and blue 50 μmol m^-2^ s^-1^) was adopted as the control treatment, providing it continuously throughout a 16 h d^-1^ photoperiod. In contrast to the control, three dynamic lighting regimes were compared, where the daily blue (B) light integral was reduced by 25%, 38%, or 50% (25% B, 38% B, and 50% B, respectively, **Table 1**), as compared to the control blue photon flux. To maintain the total PPFD in dynamic treatments constant at 200 μmol m^-2^ s^-1^, the reduction in blue photon flux was compensated by a proportional increase in red photon flux. While the control supplied a constant RB3 throughout the photoperiod, in the dynamic treatments, RB3 was periodically modified by reducing blue light with hourly alternation. In particular: (1) the 25% B treatment hourly alternated the RB3 with 175 μmol m^-2^ s^-1^ of red and 25 μmol m^-2^ s^-1^ of blue; (2) the 38% B treatment adopted a stepwise pattern in the blue light reduction, reducing it in four hourly periods as reported in **Table 1** (step 1 control RB3, step 2 162/38, step 3 175/25, and step 4 188/12 μmol m^-2^ s^-1^ of red/blue radiation, respectively); (3) the 50% B, hourly alternated the RB3 with the 200 μmol m^-2^ s^-1^ of monochromatic red light. A graphic representation of the experimental treatments is shown in **Supplementary Figure 1**.

### 2.3 Morphological measurements and growth analysis components

Plants were harvested at 7, 14, and 21 DAT. Five plants per replicate were sampled, resulting in 15 plants per treatment. Shoot fresh weight (g FW plant^-1^) was recorded at each harvest, while roots were not monitored as they remained embedded in the compacted substrate and were therefore excluded from the analysis. The fresh yield (kg FW m^-2^) was determined by scaling the average plant fresh weight to the planting density used in the experiment (140 plants m^-2^). Shoot dry weight (g DW plant^-1^) was measured after drying samples at 65 °C for 72 h, and the dry matter content (DMC, %) was calculated as the ratio of dry to fresh shoot weight. Plant height (cm) was measured from the collar to the apex using a ruler. Leaf area (cm² plant^-1^), excluding cotyledons, was determined from digital images analyzed with the Easy Leaf Area smartphone application (University of California, USA) (Easlon and Bloom, 2014), and used to calculate the leaf area index (LAI, m² m^-2^). Specific leaf area (SLA, cm² g^-1^ leaf DW) was calculated as the ratio of total leaf area to leaf dry weight, while the leaf weight ratio (LWR, g leaf g^-1^ DW) was determined as the ratio between leaf dry weight and total shoot dry biomass. Similarly, the leaf area ratio (LAR, cm² g^-1^ DW) was calculated by the ratio of the leaf area (cm^2^) to total shoot dry biomass.

To evaluate the effect of dynamic versus constant lighting at different growth stages of the cycle, relative growth rate (RGR, g DW g^-1^ DW d^-1^, **Equation 1**) and net assimilation rate (NAR, g DW cm^-2^ leaf d^-1^, **Equation 2**) were calculated according to the formula by Williams (1946), as reported below:

(1) $$\text{RGR}=\frac{\text{ln}DW_{2}-\text{ln}DW_{1}}{t_{2}-t_{1}}$$

(2) $$\text{NAR}=\frac{DW_{2}-DW_{1}}{t_{2}-t_{1}}\;\;\frac{\text{ln}LA_{2}-\text{ln}LA_{1}}{LA_{2}-LA_{1}}$$

Where DW_2_ and DW_1_, and LA_2_ and LA_1_, represent the dry weights and leaf area, respectively, at the beginning (t_1_) and at the end (t_2_) of the growing period. Although the destructive harvesting measurements were performed every 7 days, specifically at 0, 7, 14, and 21 DAT, the RGR and NAR were calculated only once along the entire crop growing period from 0 to 21 DAT to assess the effect of lighting strategies on the overall crop growth performances (Carotti et al., 2024).

### 2.4 Resource use efficiency

Light-energy use efficiency (L-EUE, g FW kWh^-1^) was calculated as the ratio of fresh yield to cumulative lamp electricity consumption (kWh m^-2^) for each spectral combination recipe at each harvest (7, 14, and 21 DAT). First, cumulative lamp electricity consumption was derived from three-day monitoring using power meters installed to each lighting treatment. Subsequently, daily energy consumption per unit surface area (kWh m^-2^ d^-1^) was calculated and reported in **Table 1**, and the cumulative energy consumption (kWh m^-2^) was calculated at each harvest period (7, 14, 21 DAT) and used for the L-EUE calculation. Similarly, light use efficiency (LUE, g DW mol^-1^) was determined as the ratio of dry weight to cumulative incident photosynthetic photons during each time period between transplanting and harvests (7, 14, and 21 DAT). Cost efficiency was expressed as production cost per kilogram of fresh yield (€ kg^-1^ FW), calculated by dividing the total lighting expense per square meter, based on a Sweden wholesale electricity price of 0.036 € kWh^-1^ (FfE, 2024), by the corresponding fresh yield. Lighting-energy consumption was recorded only during the production stage to focus the comparative analysis between treatments. Land-surface use efficiency (SUE, kg FW m^-2^ year^-1^) was evaluated by determining the potential yield, defined as the total fresh shoot weight (excluding roots and cotyledons), harvested from the experimental cultivation area of 0.5 m² per treatment. Based on the crop cycle durations of 7, 14, and 21 DAT, the number of growing cycles per year were calculated, corresponding to 49, 25, and 17 cultivation cycles year^-1^, respectively. All values were normalized to the cultivated surface area and expressed as kg of fresh weight m^-2^ year^-1^ (SUE, kg FW m^-2^ year^-1^). A half-day interval between cycles was included to account for harvesting, maintenance (including cleaning and sanitation of the system), and sowing for the new growing cycle.

### 2.5 Relative chlorophyll content

The relative chlorophyll content of leaves was estimated on the day of harvest at 7, 14, and 21 DAT using a handheld leaf chlorophyll meter (SPAD-502, Konica Minolta, Tokyo, Japan) by taking three measurements on each of the two most developed leaves (Pennisi et al., 2020).

### 2.6 Experimental design and statistical analysis

The experiment was conducted using a completely randomized design (CRD) within the ebb-and-flow cultivation sector, with four lighting treatments, each replicated three times in separate compartments and assigned randomly (**Supplementary Figure 2**). At each harvest time (7, 14, and 21 DAT), measurements were taken on 15 plants per treatment. To minimize edge effects and maintain consistent planting density, harvested plants were replaced with border or buffer plants, not used for further measurements. Prior to analysis, data were tested for normality and homogeneity of variance. Variables meeting these assumptions were analyzed using one-way ANOVA, followed by Tukey’s *post hoc* test at a 5% significance level (p < 0.05). For variables that did not meet normality assumptions, data were transformed using the orderNorm function from the bestNormalize package (Peterson and Cavanaugh, 2020) to achieve normality. This approach was applied to plant height (7 DAT), production cost (7 and 21 DAT), and chlorophyll content (14 DAT). While statistical significance was determined using the transformed data, all figures and tables present the back-transformed (original) means and standard errors for ease of biological interpretation. This choice also ensured consistent representation across harvest time points, as these variables required transformation only at specific stages of the growing cycle. All statistical analyses were performed in RStudio (version 2.2.2) using the agricolae package, while graphs were prepared using Microsoft Excel (version 16.16.27).

## 3 Results

### 3.1 Effect of constant and dynamic lighting on biomass accumulation

**Figure 2:**
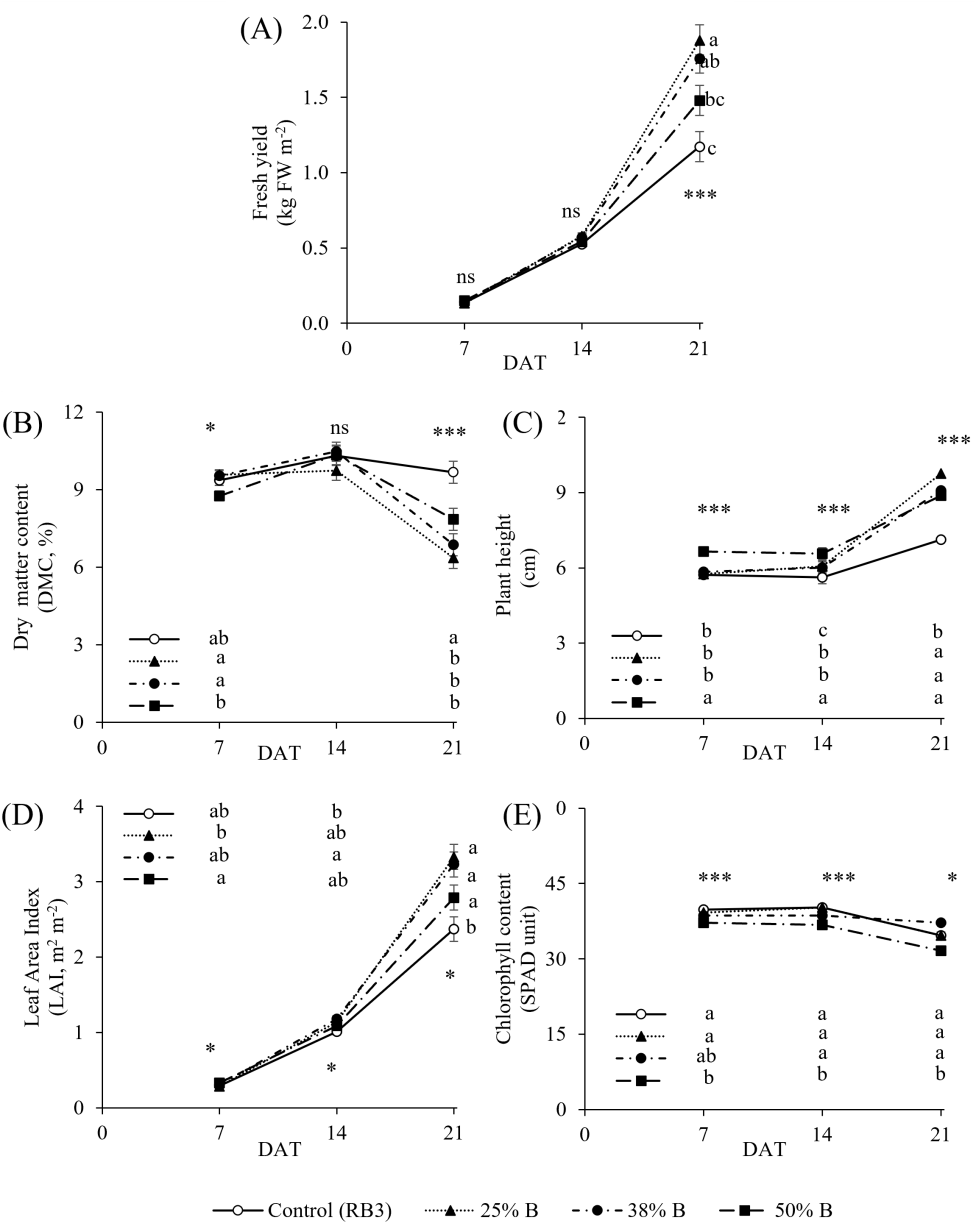
Effect of different lighting treatments on **(A)** fresh yield (kg FW m^-2^), **(B)** dry matter content (DMC, %), **(C)** plant height (cm), **(D)** leaf area index (LAI, m^2^ m^-2^), and **(E)** chlorophyll content (SPAD unit) at different harvest times (7, 14, and 21 days after transplanting, DAT). Different letters indicate significant differences among treatments within each time point, while asterisks denote significant differences (*p ≤ 0.05 and ***p ≤ 0.001), and ns indicate “not significant”. Values represent means ± standard error.

**Figure 2** illustrates the effect of constant or dynamic lighting on lettuce agronomic (yield and DMC), morphological (plant height and leaf area index), and physiological (chlorophyll content) parameters at different harvest times (7, 14, and 21 DAT). At the early growth stage (7 DAT), fresh yield (kg FW m^-2^) was not significantly affected by any of the tested lighting treatments (**Figure 2A**). A similar trend was observed for individual fresh and dry weights, averaging 1.01 g FW plant^-1^ and 0.09 g DW plant^-1^, respectively (**Supplementary Table 1**). However, dry matter content (DMC) was higher under the 25% B and 38% B dynamic lighting treatments (average 9.5%), while the 50% B dynamic treatment resulted in the lowest DMC (8.7%) (**Figure 2B**). The control (constant RB3) treatment showed intermediate DMC values, but differences against dynamic treatments were not statistically significant at 7 DAT (**Figure 2B**). At 14 DAT, no significant differences were detected among lighting treatments for fresh yield (**Figure 2A**), DMC (**Figure 2B**), plant fresh and dry weight (**Supplementary Table 1**), with average values of 0.55 kg FW m^-2^, 10%, 4.26 g FW plant^-1^, and 0.40 g DW plant^-1^, respectively. Nevertheless, from 14 to 21 DAT, the greatest increase in fresh yield occurred under the 25% B dynamic lighting, with a 223% gain, while the control increased only by 122% (**Figure 2A**). At 21 DAT, 25% B achieved the highest yield (1.88 kg FW m^-2^), significantly greater than the control and 50% B treatments by approximately 1.6- and 1.3-fold, respectively (**Figure 2A**). Dry weight followed a similar trend, although no significant differences were detected among the constant or dynamic lighting treatments (average 0.81 DW plant^-1^) at the final harvest (**Supplementary Table 1**). Between 7 and 21 DAT, DMC decreased by 33% under dynamic lighting, whereas it increased by 3% under constant RB3 lighting, resulting in a 1.37-fold increase over the dynamic treatments at the final harvest (**Figure 2B**).

### 3.2 Effect of constant and dynamic lighting on plant morphological response

Plants grown under 50% B dynamic lighting were significantly taller throughout the growth stages compared to the control treatment (7.12 cm), reaching 8.8 cm at the final harvest (**Figure 2C**). Notably, at the initial stage (7 DAT), the height of the control plants was comparable to those in the 25% B and 38% B treatments, which were significantly shorter than the 50% B treatment (**Figure 2C**). However, in the subsequent harvests (14 DAT and 21 DAT), the control consistently exhibited a more compact plant height than the dynamic lighting treatments (**Figure 2C**). At the early growth stage (7 DAT), the highest LAI was recorded under the 50% B light treatment (LAI, 0.33 m^2^ m^-2^), while the 25% B treatment showed the lowest value (LAI, 0.28 m^2^ m^-2^); the control and 38% B treatments remained statistically comparable to the others (**Figure 2D**). At 14 and 21 DAT, the 38% B dynamic lighting produced significantly higher LAI (1.2 and 1.4-fold, respectively) than the control (which featured LAI of 1.1 and 2.37 m^2^ m^-2^, **Figure 2D**), and the 25% B also outperformed the control at 21 DAT in LAI development. Overall, no significant differences in LAI were observed between the constant RB3 lighting and 50% B dynamic treatment throughout the growth cycle (**Figure 2D**).

### 3.3 Effect of constant and dynamic lighting on leaf chlorophyll content

Considering relative chlorophyll content, the control treatment consistently maintained higher SPAD levels throughout the crop cycle as compared to the 50% B dynamic lighting treatment, which showed the lowest values (**Figure 2E**). Chlorophyll content in the control was 1.06, 1.09, and 1.17-fold higher than in 50% B treatment at 7, 14, and 21 DAT, respectively (**Figure 2E**). Interestingly, the dynamic treatments 25% B and 38% B did not differ significantly from the control at any of the measured growth stages (**Figure 2E**).

### 3.4 Effect of constant and dynamic lighting on growth

**Figure 3:**
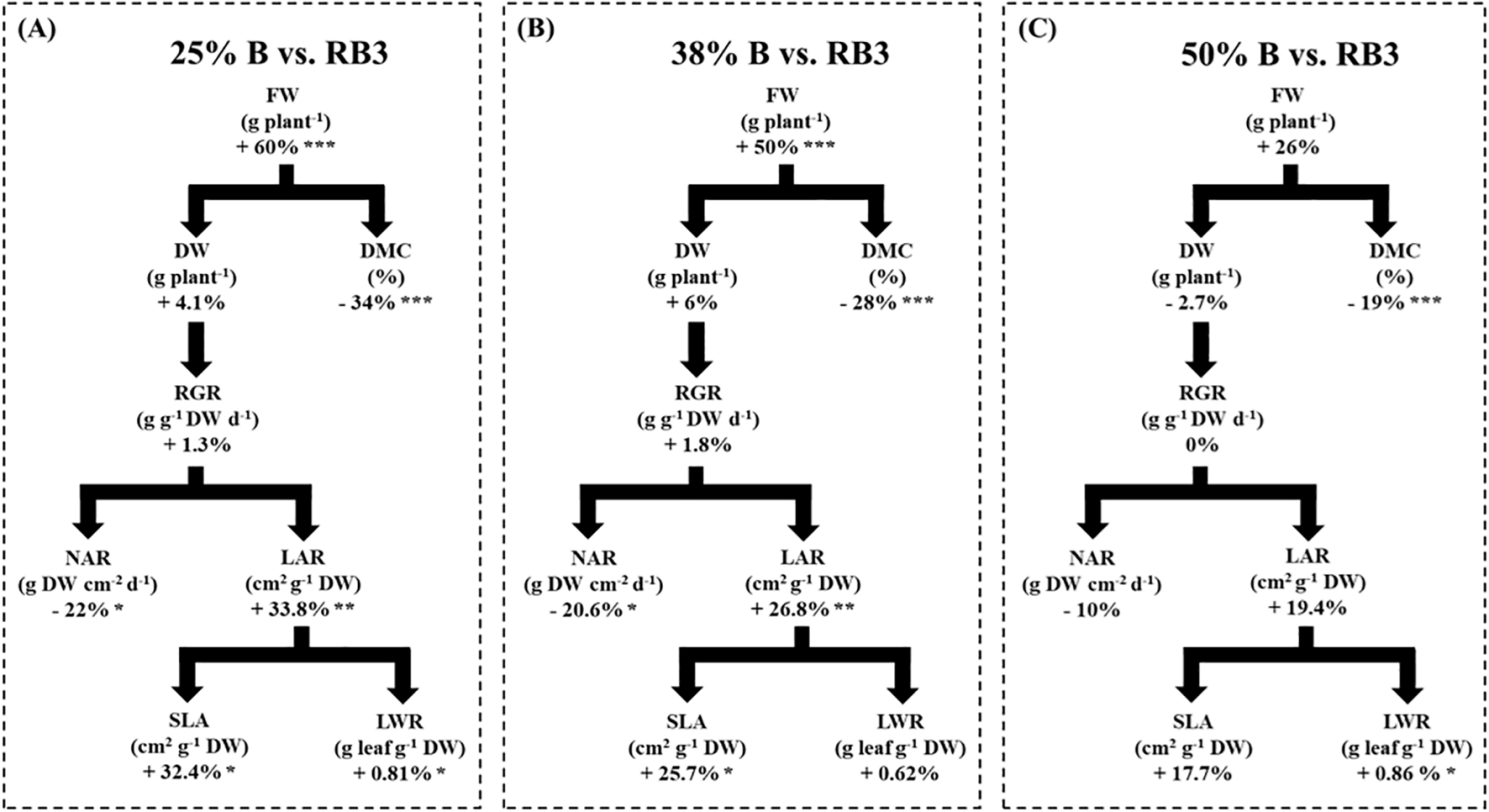
Effect of dynamic lighting strategies versus control (constant RB3) on growth components in lettuce at 21 days after transplanting. **(A)** Control RB3 vs 25% B, **(B)** Control RB3 vs 38% B, and **(C)** Control RB3 vs 50% Percentage values indicate the increase or decrease relative to control (RB3). Asterisks denote significant differences compared with RB3 according to one-way ANOVA (*p ≤ 0.05, **p ≤ 0.01, ***p ≤ 0.001). Measured parameters include fresh weight (FW, g plant^-1^), dry weight (DW, g plant^-1^), dry matter content (DMC, %), relative growth rate (RGR, g g^-1^ DW d^-1^; Formula 1), net assimilation rate (NAR, g DW cm^-2^ d^-1^; Formula 2), leaf area ratio (LAR, cm² g^-1^ DW), specific leaf area (SLA, cm² g^-1^ DW), leaf area (LA, cm² plant^-1^), and leaf weight ratio (LWR, g leaf g^-1^ DW).

At the final harvest stage (21 DAT), dynamic lighting with varying blue light fractions (25%, 38%, and 50% B) influenced the lettuce growth performance as compared with the constant RB3 control (**Figure 3**). The 25% B treatment significantly increased plant fresh weight compared to the control (**Figure 3A**), mainly attributable to greater shoot water content, as evidenced by the lack of significant differences in dry weight and the resulting significant reduction in DMC (**Figure 3A**). Because dry weight was not significantly affected by 25% B treatment, the RGR also showed no significant change under this lighting strategy (**Figure 3A**). At the same time, NAR decreased under the 25% B treatment, and LAR increased significantly, driven by a higher SLA and LWR (**Figure 3A**). A similar pattern in growth components was observed between the RB3 control and the 38% B treatment, with plant fresh weight increasing by 50%. However, in this case, the slight increase observed in LWR was not significant (**Figure 3B**). In contrast to the previous two dynamic lighting strategies, the 50% B treatment did not significantly improve the plant fresh weight, nor did it affect any other growth components (DW, RGR, NAR, LAR, and SLA). The only exceptions were the DMC, which was also reduced by 19% in the 50% B treatment, as well as the LWR, featuring a modest but significant increase of 0.86% (**Figure 3C**).

### 3.5 Effect of constant and dynamic lighting on light-energy use efficiency, production cost, and surface use

**Figure 4:**
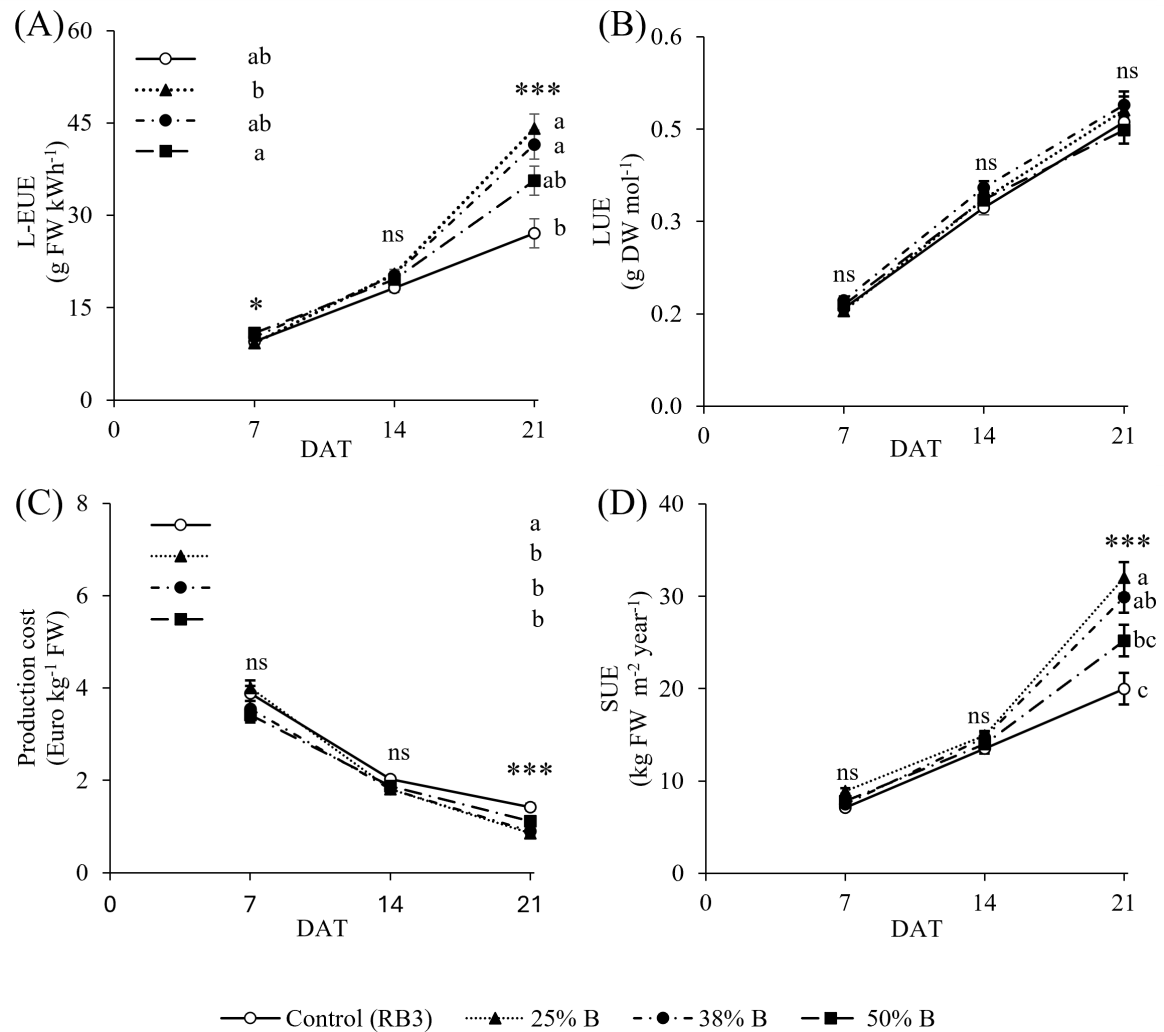
Effect of different lighting treatments on **(A)** light-energy use efficiency (L-EUE, g FW kWh^-1^), **(B)** light use efficiency (LUE, g DW mol^-1^), **(C)** production cost (€ kg^-1^ FW), and **(D)** surface use efficiency (SUE, kg FW m^-2^ year^-1^) at different harvest time (7, 14, and 21 days after transplanting, DAT). Different letters indicate significant differences among treatments within each time point for p ≤ 0.05 (*) and p ≤ 0.001 (***), while ns indicate “not significant”. Values represent means ± standard error (n = 15).

Light-energy use efficiency (L-EUE) showed significant differences between constant and dynamic lighting treatments, but only at the final harvest (**Figure 4A**). At 7 DAT, the RB3 control did not show significant differences compared to the dynamic treatments, although the 50% B treatment showed a significantly higher L-EUE (10.90 g FW kWh^-1^) compared to the 25 B% treatment where the lowest value was observed (9.26 g FW kWh^-1^; **Figure 4A**). At 14 DAT, no significant differences in L-EUE were observed among the lighting treatments, including both dynamic and constant regimes, with values remaining consistent across treatments and averaging 19.6 g FW kWh^-1^ (**Figure 4A**). At 21 DAT, the highest L-EUE was observed under the 25% B treatment (44.1 g FW kWh^-1^) and 38% B (41.5 g FW kWh^-1^), showing notable improvements over control RB3 (27.1 g FW kWh^-1^), which had the lowest L-EUE value. An intermediate L-EUE was recorded under the 50% B (35.6 g FW kWh^-1^) treatment, which did not differ significantly from other tested treatments (**Figure 4A**).

Light use efficiency (LUE) increased steadily across growth stages (**Figure 4B**) with no significant differences among treatments at any growth stage, with average values of 0.16, 0.33, and 0.47 g DW mol^-1^ at 7, 14, and 21 DAT, respectively (**Figure 4B**).

Production cost (€ kg^-1^ FW, **Figure 4C**) showed a pattern that was inverse of the trend of L-EUE (**Figure 4A**). While at 7 and 14 DAT no significant difference was observed among the tested treatments, at 21 DAT dynamic lighting treatments 25% B (0.85 € kg^-1^), 38% B (0.89 € kg^-1^), and 50% B (1.1 € kg^-1^) achieved the lowest production costs, all significantly lower than the control (1.42 € kg^-1^) by 40% or more (**Figure 4C**). There were no significant differences in surface use efficiency (SUE) in the first two growth stages (**Figure 4D**), while at the final harvest, the SUE (**Figure 4D**) mirrored the values of fresh yield (**Figure 2A**). Over the annual production cycle, dynamic lighting treatments outperformed the control one, achieving a maximum SUE of 32 kg FW m^-2^ year^-1^ under 25% B; however, the 50% blue treatment did not differ significantly from the 38% B or the control (**Figure 4D**). Overall, the greatest increase in SUE between 14 and 21 DAT was observed in the 25% B treatment, showing a 2.2-fold increase, whereas the control exhibited the lowest increase at 1.5-fold (**Figure 4D**).

## 4 Discussion

### 4.1 Dynamic lighting effect on plant growth, dry matter content, and growth indexes

The effects of light on plants are complex, as it regulates photosynthesis, growth, and various developmental processes (Wu et al., 2025). Within the light spectrum, red and blue wavelengths are strongly absorbed by chlorophylls (a and b) and regulate various physiological processes, including seed germination and leaf morphology, ultimately leading to plant development and overall yield (Luo et al., 2024). Our results have clearly shown that the effect of dynamic lighting was visible depending on the when data collection was performed (**Figure 2A**). However, individual fresh and dry weight were not significantly affected during the early growth stages (7 and 14 DAT) with respect to constant or dynamic irradiation (**Supplementary Table 1**), as similarly reported by Carotti et al. (2022) and Jin et al. (2023b), suggesting that plant responses to changes in red and blue light ratios may not be immediate but may become evident only at later stages. Indeed, at the final harvest, the highest fresh biomass production was observed in 25% B and 38% B treatments (**Figure 2A**), aligning with previous findings from Van Brenk et al. (2024) showing that higher daily blue-light fraction, applied throughout the day or during specific periods, can reduce fresh yield. However, reducing the blue by 50% (50% B treatment), did not enhance the fresh yield and was statistically comparable to the RB3 control, as supported by similar findings from Chen et al. (2021), who reported that red light promotes lettuce growth only when supplemented with sufficient blue light. This behavior might be the consequence of the hourly monochromatic red supplied during the photoperiod, likely inducing the plants grown under 50% B to red light syndrome conditions, as suggested by the lowest chlorophyll content shown in **Figure 2E** (Hogewoning et al., 2010). The lower chlorophyll content under limited blue light supply (50% B) is consistent with previous findings (Snowden et al., 2016; Fan et al., 2013), underscoring the critical role of blue light in maintaining chlorophyll biosynthesis.

Considering that dry biomass accumulation was unaffected by spectral conditions (**Supplementary Table 1**), the fresh weight increase observed under the dynamic regimes (**Figure 2A**) results from changes in tissue water status rather than enhanced carbon assimilation, as confirmed by the lower DMC (**Figure 3**). The role of red and blue light in stomatal opening has been extensively reviewed by Matthews et al. (2020), who highlight their direct involvement in different physiological pathways. While red light coordinates stomata opening with photosynthesis to meet CO_2_ demand, the blue light mechanism is considered independent of carbon assimilation, leading to a supplemental increase in stomatal conductance even if photosynthesis is saturated by red light (Matthews et al., 2020). Based on that, we can argue that in our experiment, the constant RB3, which supplied the highest level of blue light, might have experienced a constantly higher stomatal conductance during the day as compared with the dynamic lighting treatments, where the shift to low blue light supply might have intermittently reduced the stomatal opening. Although we are aware that this should be confirmed with physiological measurements (transpiration and stomatal conductance), this hypothesis is supported by the findings of Zait et al. (2017), who observed reductions in transpiration and stomatal conductance in banana when the red-blue spectrum was shifted to a monochromatic red. We hypothesize that the light shift from high to low blue light availability might have altered the cell turgor relations in 25% B and 38% B: during the stomata closure, cell turgor increases due to high water flux into the xylem determined by the high blue conditions before the sudden light shift, driving leaf expansion resulting in higher SLA (**Figure 3A**). However, this hypothesis does not align with the findings of Zait et al. (2017), who reported higher turgor pressure in plants grown under constant monochromatic blue light than under monochromatic red light, although the authors did not measure turgor pressure in a shifting lighting environment. The lack of studies on the cell turgor pressure after shifting red-blue lighting still limits our understanding of the physiological process behind the higher fresh yield observed under 25% B and 38% B, therefore, we recommend further research to clarify the plant water relations under shifting lighting. Notwithstanding, this hydration advantage has direct commercial impact: leafy greens (95% water content) are market-priced by fresh biomass, so dynamic lighting could improve revenue efficiency even without additional carbon gain. However, excessive hydration could potentially reduce shelf life, which should be evaluated in future experiments.

In our experiment, dynamic lighting also influenced the plant morphology. Plant height was reduced under constant RB3 irradiation compared with the dynamic treatment (**Figure 2C**), resulting in a more compact canopy architecture. Similar responses have been reported previously by Snowden et al. (2016) who showed that increasing blue light significantly reduced stem length in tomato, cucumber, and pepper at higher irradiance levels, while Naznin et al. (2019) reported progressive reductions in plant height of lettuce and kale as the proportion of blue light increased under red-dominant spectra, highlighting the key role of spectral composition in controlling plant morphology. Furthermore, at the final harvest (21 DAT), LAI was significantly lower under constant RB3 lighting compared to the dynamic 25% B and 38% B treatments, but not under 50% B (**Figure 2D**). The effect of blue light has already been well documented on canopy structure, with high blue light supply being responsible to reduce leaf expansion in lettuce, and consequently, lower LAI (Van Brenk et al., 2024; Pennisi et al., 2019).

Our findings clearly show that the growth components were affected by dynamic lighting (**Figure 3**). The RGR integrates two key components of plant growth: LAR (the product of SLA and LWR, which reflects canopy investment) and NAR (representing photosynthetic efficiency per unit leaf area) (Medek et al., 2007). Lighting treatments did not significantly affect RGR at the final harvest in our study (**Figure 3**). Similarly, Jadhav et al. (2025) reported that a consistent growth rate has been observed in lettuce across different environmental conditions (i.e., planting density), reflecting a species-specific adaptive mechanism that maintains overall biomass accumulation efficiency. Conversely, NAR was drastically decreased in 25% B and 38% B dynamic treatments compared to constant lighting (**Figures 3A, B**), indicating a potential shift in resource allocation, consistent with growth analysis concepts (Poorter et al., 2012). The plant compensated for the lower photosynthetic efficiency per leaf area (NAR) by significantly increasing its LAR, supported by the higher LAI values observed (**Figure 2D**) and, more specifically, by the higher SLA (**Figure 3**), indicating greater leaf expansion per unit mass. Although the LWR was also significantly affected by dynamic lighting, its contribution to the LAR is negligible (**Figure 3**). Supporting these findings, previous studies have shown that higher level of blue light (such as in RB3, as compared with dynamic lighting strategies) often promotes the development of thicker, more photosynthetically efficient leaves, even as overall leaf area or biomass growth may not increase significantly. For example, Hogewoning et al. (2010) found that increasing blue light fraction enhanced the maximum photosynthetic capacity in cucumber, attributed to structural changes in chloroplasts and leaf anatomy. Similarly, Wang et al. (2024a) reported that blue light increased specific leaf weight and leaf thickness in cucumber. At the same time, total biomass did not necessarily rise, indicating a shift toward denser, more efficient leaves rather than overall growth acceleration. These morphological changes often help plants optimize light capture and carbon assimilation under varying light environments. This adaptive physio-morphological response under dynamic light regimes maintained growth rates while maximizing market-relevant fresh biomass by enhancing leaf water content.

### 4.2 Dynamic red–blue light modulation improves resource use reducing cost of production

Since artificial lighting is the primary energy input in VFs, optimizing lighting efficiency is crucial for minimizing operational costs. A lighting system’s effectiveness depends on both the distribution of spectral energy and the efficiency with which electrical energy is converted into light (Kozai, 2013). Therefore, improving energy efficiency requires understanding how the dynamic ratio of red and blue light influences both plant growth and energy use. In this study, the dynamic lighting treatment with 50% B significantly enhanced lighting-energy use efficiency (L-EUE) during early growth (7 DAT) compared to the 25% B treatment (**Figure 4A**), supporting a sustained plant growth (**Figure 2A**) with lower energy use (**Table 1**). By the final harvest (21 DAT), the advantage of the dynamic 50% B lighting strategy had diminished, becoming comparable to that of constant RB3 lighting. In contrast, the 25% B dynamic treatment increased L-EUE by 1.6-folds compared to the control, emphasizing red light’s critical role in enhancing energy efficiency (**Figure 4A**). These results are consistent with previous findings. Although red light is energetically more efficient, incorporating 10–25% blue light maximizes overall lighting efficiency and plant performance (Pennisi et al., 2019). Similarly, another study showed that LUE and EUE peaked at 90% red and 10% blue, whereas efficiency decreased below 100% red, demonstrating that even a small fraction of blue is essential to fully realize the energetic benefits of red light (Chen et al., 2021). Their finding parallels our results, which show that the 50% B treatment did not substantially enhance the L-EUE at the final harvest, as it was probably penalized by the absence of blue light during monochromatic red periods throughout the day.

Light use efficiency (LUE) in lettuce strongly depends on cultivation conditions. VFs-grown plants achieving up to 0.55 g DW mol^-1^, compared to 0.39 g mol^-1^ in greenhouses and 0.23 g mol^-1^ in the field (Jin et al., 2023a). It is also estimated that VFs can approach the theoretical maximum LUE (ranging from 1.26 to 1.81 g DW mol^−1^) due to precise control of environmental factors, making them highly efficient for lettuce production (Chen et al., 2021). In our experiment, LUE was not influenced by constant or dynamic irradiation; however, it increased progressively from 0.16 to 0.5 g DW mol^-1^ by the final harvest (21 DAT; **Figure 4B**). Similarly, Jadhav et al. (2025) reported a LUE value of 0.45 g DW mol^-1^ in lettuce under comparable lighting conditions at 29 days after sowing.

Production costs were more than 40% lower with any dynamic lighting treatment than with constant RB3 lighting (0.85 vs 1.42 € kg^-1^ FW; **Figure 4C**), demonstrating the potential for energy savings from spectral scheduling. Because economic performance is directly linked to fresh biomass yield, dynamically adjusting the red–blue ratio may help maintain, or even increase, productivity while reducing electricity consumption.

### 4.3 Dynamic red–blue light modulation maximizes land-surface use efficiency

Finally, land or surface-use efficiency (SUE) is a key factor in enhancing food security and addressing the limitations of traditional agriculture. In the VFs facility, cultivation occurs in the vertical dimension, making it highly efficient in terms of production per required area (Orsini and Zauli, 2023). In our experiment, SUE reflected the crop fresh yield (**Figure 2A**), with the 25% B dynamic lighting treatment producing 32 kg FW m^-2^ year^-1^, a 60% increase compared to the constant RB3 control (20 kg FW m^-2^ year^-1^, **Figure 4D**). Our findings fall within the wide range of lettuce productivity of research settings reviewed by Pennisi et al. (2025), centered on a median of 48.3 kg m^-2^ year^-1^. Similarly, Zauli et al. (2024) reported that intermediate red:blue ratios (e.g., RB 5) optimized baby-leaf kale yield and SUE up to 54 kg FW m^-2^ year^-1^. In our experiment, the SUE between 14 and 21 DAT increased by 114% under dynamic 25% B light, compared to just 48% in the constant (RB3) treatment, indicating the advantage of dynamically managed blue and red lighting spectrum to inform scalable production models. Future optimization should refine harvest timing, as the drastic increase in SUE between 14 and 21 DAT indicates a critical window for maximizing yield per unit area.

## 5 Conclusion

This study provides novel insights into enhancing sustainability in vertical farming systems by examining the effects of dynamic versus constant lighting on lettuce growth, morphology, physiology, and resource-use efficiency. We demonstrated that it is possible to reduce blue light supply in lettuce cultivation and thus lighting energy use by dynamically adjusting red and blue lighting based on hourly changes during the photoperiod. While reducing the blue light by 50% did not improve the overall crop performances as compared to the constant RB3, the reduction of blue light by 25-38% has increased the crop fresh yield, which in turn improved the lighting energy use efficiency and reduced the lighting cost by 40%, making them scalable strategies for commercial vertical farming.

In our experiment, the enhanced fresh yield under dynamic lighting was associated with coordinated morphological and physiological adjustments, resulting in higher plant water content, underscoring the influence of dynamic lighting on plant water relations. However, the lack of physiological measurements (e.g., transpiration, stomatal conductance, and photosynthesis) is a key limitation of our work, preventing us from clarifying the role of dynamic red-blue lighting on plant water relations. Future studies should shed light on the mechanisms underlying higher water uptake by plants under dynamic lighting and their influence on crop water-use efficiency. Furthermore, future work should integrate life cycle assessment (LCA) and life cycle costing (LCC) approaches to evaluate the full environmental and economic impacts of dynamic lighting across different crop species. Moreover, coupling spectral management with environmental control parameters (e.g., temperature, air flow, humidity, and CO_2_) will be essential to optimize overall system efficiency. Such interdisciplinary optimization may inform the future development of low-energy and resource-efficient VFs systems for sustainable urban food production.

## Data Availability

The raw data supporting the conclusions of this article will be made available by the authors, without undue reservation.
