# Supplementary material for: Optimizing growth efficiency and energy economics in vertical farming through dynamic reduction of blue light in lettuce baby leaf (Lactuca sativa L.)
Source: Front Plant Sci. 2026 Apr 22;17:1827422. doi: 10.3389/fpls.2026.1827422 (PMC13143623; doi:10.3389/fpls.2026.1827422)
Supplement: Supplementary file 1 [file Supplementaryfile1.docx]

# Supplementary Material

**Supplementary Figure 1**. Graphic representation of the experimental treatments. Note the 16 h photoperiod from 06:00 to 22:00, and the hourly red and blue adjustments maintaining 200 μmol m^-2^ s^-1^ as total photosynthetic photon flux density.


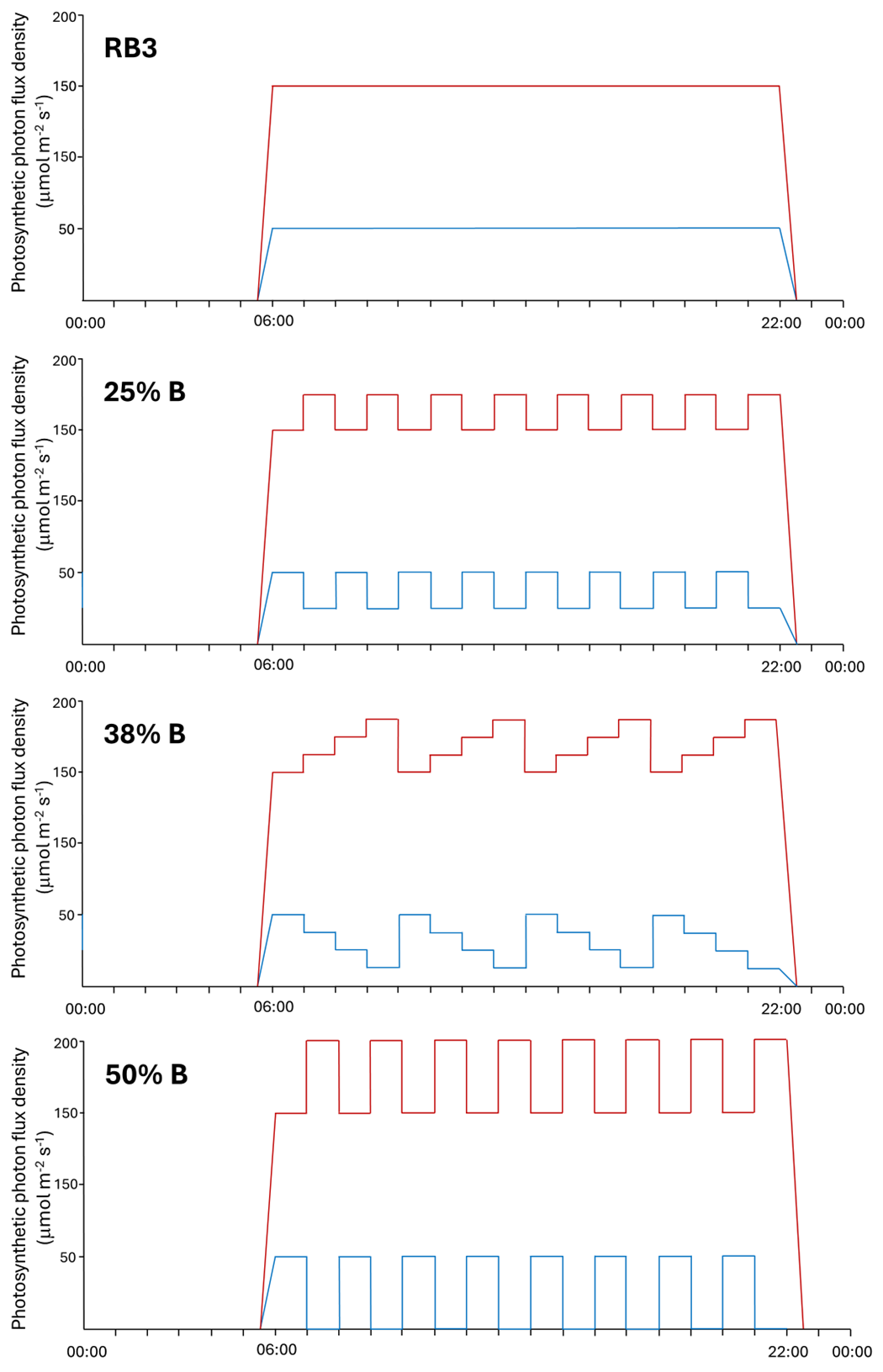


**Supplementary Figure 2**. Schematic layout of the experimental design, arranged as a completely randomized design, conducted within Nära Sverige AB vertical farm, Stockholm, Sweden. The experiment composed with four lighting treatments with each of three replications. Twelve light-insulated compartments, each measuring 0.5 m², were constructed for this study.


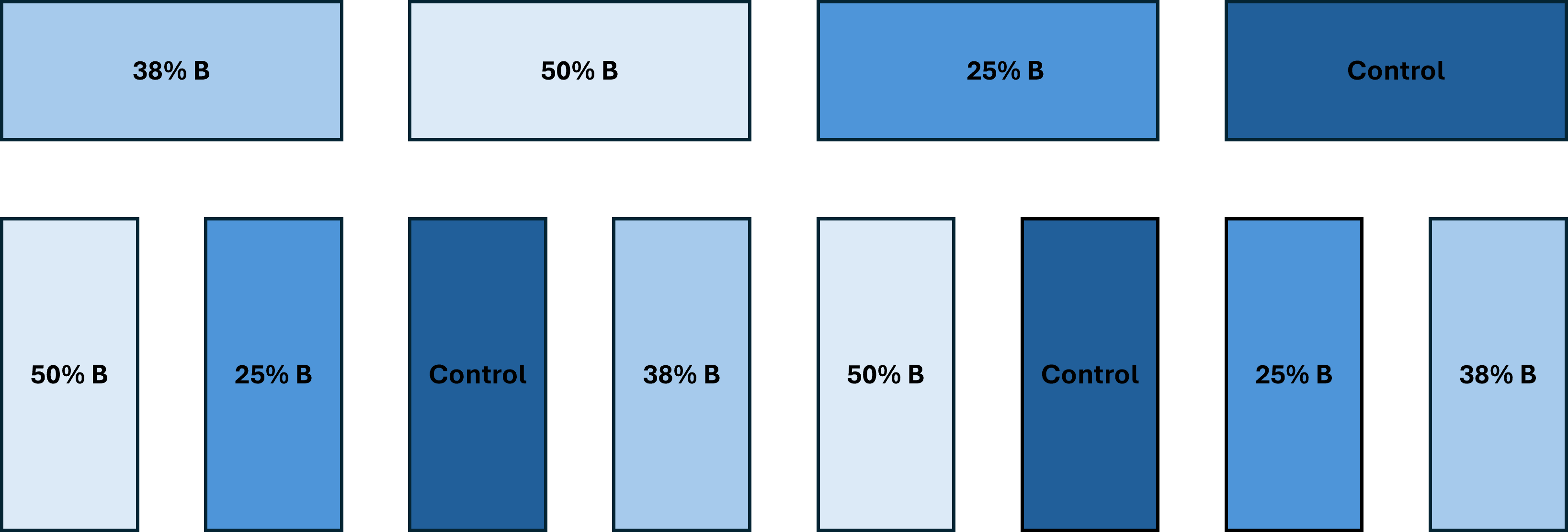


**Supplementary Table 1**. Effect of dynamic versus constant lighting treatments on agronomic parameters at different harvest time (7, 14, and 21 days after transplanting, DAT).

| **Treatments** | **Parameters** | |
| --- | --- | --- |
|  | Fresh weight (g FW plant^-1^) | Dry weight (g DW plant^-1^) |
| 7 days after transplanting (DAT) | | |
| Control | 0.98 a | 0.091 a |
| B 25% | 0.94 a | 0.089 a |
| B 38% | 1.04 a | 0.099 a |
| B 50% | 1.08 a | 0.094 a |
| 14 days after transplanting (DAT) | | |
| Control | 3.76 a | 0.382 a |
| B 25% | 4.15 a | 0.399 a |
| B 38% | 4.08 a | 0.423 a |
| B 50% | 3.89 a | 0.398 a |
| 21 days after transplanting (DAT) | | |
| Control | 8.39 c | 0.798 a |
| B 25% | 13.44 a | 0.831 a |
| B 38% | 12.58 ab | 0.846 a |
| B 50% | 10.58 bc | 0.776 a |
